# Biochemical Characterization of QPX7728, a New Ultrabroad-Spectrum Beta-Lactamase Inhibitor of Serine and Metallo-Beta-Lactamases

**DOI:** 10.1128/AAC.00130-20

**Published:** 2020-05-21

**Authors:** Ruslan Tsivkovski, Maxim Totrov, Olga Lomovskaya

**Affiliations:** a Qpex Biopharma Inc., San Diego, California, USA; b Molsoft L.L.C., San Diego, California, USA

**Keywords:** QPX7728, beta-lactamase inhibition, kinetics, metallo-beta-lactamase, serine beta-lactamase

## Abstract

QPX7728 is a new ultrabroad-spectrum inhibitor of serine and metallo-beta-lactamases (MBLs) from a class of cyclic boronates that gave rise to vaborbactam. The spectrum and mechanism of beta-lactamase inhibition by QPX7728 were assessed using purified enzymes from all molecular classes. QPX7728 inhibits class A extended-spectrum beta-lactamases (ESBLs) (50% inhibitory concentration [IC_50_] range, 1 to 3 nM) and carbapenemases such as KPC (IC_50_, 2.9 ± 0.4 nM) as well as class C P99 (IC_50_ of 22 ± 8 nM) with a potency that is comparable to or higher than recently FDA-approved beta-lactamase inhibitors (BLIs) avibactam, relebactam, and vaborbactam. Unlike those other BLIs, QPX7728 is also a potent inhibitor of class D carbapenemases such as OXA-48 from *Enterobacteriaceae* and OXA enzymes from Acinetobacter baumannii (OXA-23/24/58, IC_50_ range, 1 to 2 nM) as well as MBLs such as NDM-1 (IC_50_, 55 ± 25 nM), VIM-1 (IC_50_, 14 ± 4 nM), and IMP-1 (IC_50_, 610 ± 70 nM). Inhibition of serine enzymes by QPX7728 is associated with progressive inactivation with a high-efficiency *k*_2_/*K* ranging from 6.3 × 10^4^ (for P99) to 9.9 × 10^5^ M^−1^ s^−1^ (for OXA-23). This inhibition is reversible with variable stability of the QPX7728-beta-lactamase complexes with target residence time ranging from minutes to several hours: 5 to 20 min for OXA carbapenemases from A. baumannii, ∼50 min for OXA-48, and 2 to 3 h for KPC and CTX-M-15. QPX7728 inhibited all tested serine enzymes at a 1:1 molar ratio. Metallo-beta-lactamases NDM, VIM, and IMP were inhibited by a competitive mechanism with fast-on–fast-off kinetics, with *K_i_*s of 7.5 ± 2.1 nM, 32 ± 14 nM, and 240 ± 30 nM for VIM-1, NDM-1, and IMP-1, respectively. QPX7728’s ultrabroad spectrum of BLI inhibition combined with its high potency enables combinations with multiple different beta-lactam antibiotics.

## Abstract (precis)

QPX7728 is a new ultrabroad-spectrum inhibitor of serine and metallo-beta-lactamases (MBLs) from a class of cyclic boronates that gave rise to vaborbactam. The spectrum and mechanism of beta-lactamase inhibition by QPX7728 were assessed using purified enzymes from all molecular classes. QPX7728 inhibits class A extended-spectrum beta-lactamases (ESBLs) (50% inhibitory concentration [IC_50_] range, 1 to 3 nM) and carbapenemases such as KPC (IC_50_, 2.9 ± 0.

## INTRODUCTION

Beta-lactam antibiotics belong to four major classes, penicillins, cephalosporins, monobactams, and carbapenems; collectively, they are the most widely used group of antimicrobial agents for treatment of bacterial infections, in both community (oral penicillins and oral cephalosporins) and hospital (penicillins, cephalosporins, monobactams, and carbapenems) settings owing to their broad-spectrum nature, bactericidal mode of action, and excellent safety profile (1, 2). However, resistance to all types of beta-lactams has emerged in the United States (3) and worldwide (4), limiting the utility of this important class of antibiotics. The most significant mechanism that mediates resistance to beta-lactams is the production of beta-lactamases, inactivating enzymes that hydrolyze the amide bond of the beta-lactam ring (5).

These diverse beta-lactamases are grouped into four molecular classes based on sequence and structural similarity (6, 7). Beta-lactamases that belong to classes A, C, and D are the so-called serine enzymes (SBLs); the nucleophilic serine in their active center is required for the hydrolytic reaction. Beta-lactamases from class B require Zn(II) for their activity and thus are metalloenzymes (MBLs). MBLs are divided into three subclasses, B1, B2, and B3, based on sequence similarity (8). Beta-lactamases from different molecular classes often have overlapping substrate specificity (7).

Development of beta-lactamase inhibitors (BLIs) in combination with beta-lactams is a powerful strategy to protect beta-lactams from beta-lactamase-mediated hydrolysis, thus extending their clinical utility. The first three BLIs that reached the clinic in the 1980s and 1990s, clavulanic acid, tazobactam, and sulbactam, are all beta-lactam-based molecules that are irreversible suicidal inhibitors. They inactivate beta-lactamases in a stepwise process that involves the opening of the beta-lactam ring of the BLIs and its irreversible covalent binding to the active site serine. These BLIs have a limited spectrum that is restricted mainly to class A penicillinases (TEM-1 and SHV-1) and extended-spectrum beta-lactamases (ESBLs) from SHV, TEM, and CTX-M subfamilies (9). They do not inhibit the class A carbapenemase KPC because KPC can efficiently hydrolyze these beta-lactam-type molecules in the same way it does beta-lactam antibiotics (10).

**FIG 1:**
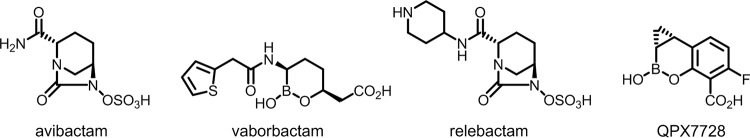
Beta-lactamase inhibitors.

Three newer BLIs that are now in clinical use, avibactam (approved by the FDA in 2015 in combination with ceftazidime), vaborbactam (approved by the FDA in 2017 in combination with meropenem), and relebactam (approved by the FDA in 2019 in combination with imipenem), are all derived from non-beta-lactam scaffolds (Fig. 1) (11). Avibactam and relebactam are diazabicyclooctane derivatives (DBOs), while vaborbactam is based on a cyclic boronic acid pharmacophore (12). These three BLIs are dual inhibitors of class A and class C beta-lactamases, and all three are potent inhibitors of KPC. Avibactam (but not relebactam or vaborbactam) can also inhibit some class D enzymes (13). Similarly to clavulanic acid and tazobactam, beta-lactamase inhibition by avibactam (and other DBOs) involves covalent binding of the opened ring inhibitor to the active site serine (14, 15). However, unlike beta-lactam-based BLIs, this binding is slowly reversible due to recyclization of avibactam which in most cases is followed by the release of intact enzyme and intact avibactam. The exception is KPC, which can slowly desulfate the avibactam adduct, resulting in slow inactivation of avibactam (14) (and other DBOs [16]). As this inactivation is very slow, it apparently does not affect antibiotic potentiation activity of avibactam and other DBOs.

The mechanism of inhibition of beta-lactamases by vaborbactam is also based on a reversible covalent binding where the boronate of vaborbactam makes a covalent bond with the active site serine, forming a tetrahedral intermediate and functioning as a transition state analog (12). This process does not involve the opening of the vaborbactam ring; the intact compound is released from the enzyme. An appealing feature of vaborbactam is an unusually low dissociation rate of the KPC-vaborbactam complex with a target residence time of several hours (17). No degradation of vaborbactam by KPC has been detected (17).

Combination agents recently approved by the FDA based on the three BLIs described above represent significant progress in addressing serious drug-resistant Gram-negative bacterial infections, particularly those caused by KPC-producing carbapenem-resistant *Enterobacteriaceae*; however, none of these BLIs inhibit metallo-beta-lactamases; hence, the combination agents are not active against metallo-beta-lactamase-producing carbapenem-resistant *Enterobacteriaceae* (CRE). Likewise, since none of these BLIs inhibit class D carbapenemases in Acinetobacter baumannii, these agents do not have utility against this important pathogen (18). Of note, none of these agents have oral formulations available for the treatment of infections due to serious or urgent resistant threats in setting where intravenous (i.v.) therapy is not available or desirable (19).

Prompted by this ongoing unmet clinical need, we initiated a program that involved modifications to the boronic acid pharmacophore with a goal of expanding the spectrum of beta-lactamase inhibition to include both serine and metallo-beta-lactamases and to achieve oral bioavailability. QPX7728, which has properties that achieved our preclinical targets in a single molecule, was discovered. QPX7728 is an ultrabroad-spectrum inhibitor with activity against numerous serine and metallo-beta-lactamases, including carbapenemases such as class A KPC, class B NDM and VIM, and class D OXA-48 and OXA-23 that are found in carbapenem-resistant *Enterobacteriaceae* and in carbapenem-resistant Acinetobacter baumannii, respectively. In addition, QPX7728 can be delivered by oral administration (20); thus, the ultrabroad-spectrum beta-lactamase inhibition spectrum could be applied to i.v. and oral QPX7728-based combination products.

## RESULTS AND DISCUSSION

### QPX7728 is an ultrabroad-spectrum inhibitor of diverse serine and metallo-beta-lactamases.

The inhibition profile of QPX7728 (Fig. 1) (defined as 50% inhibitory concentration [IC_50_]) of inhibition of hydrolysis of nitrocefin (NCF) or imipenem (for NDM and IMP MBLs) by several purified beta-lactamases from all four molecular classes is shown in Table 1. QPX7728 inhibited class A ESBLs CTX-M-14 and CTX-M-15, SHV-12, TEM-10, and TEM-26 with IC_50_ values in a 1 to 3 nM range, a potency generally similar to avibactam and greater than relebactam or vaborbactam. QPX7728 was also a potent inhibitor of KPC-2 with an IC_50_ of ∼3 nM. IC_50_ values of KPC-2 inhibition for the comparator BLIs vaborbactam, avibactam, and relebactam ranged from 22 nM to 110 nM (Table 1). The IC_50_ for inhibition of class C beta-lactamase P99 from Enterobacter cloacae by QPX7728 was ∼22 nM and was similar to that for comparator BLIs. QPX7728 also demonstrated potent inhibition of class D carbapenemase OXA-48 with an IC_50_ of 1 nM, which is 160-fold more potent than avibactam. As reported earlier, neither relebactam (21) nor vaborbactam (17) inhibits OXA-48. Importantly, the same high potency (IC_50_ of 1 nM) was observed for QPX7728 inhibition of OXA-23, the class D carbapenemase from A. baumannii. None of the comparator BLIs had activity against this enzyme (Table 1). For vaborbactam, which relies on the favorable interactions of its amide “side chain” carbonyl (Fig. 1) for some of its potency (12), the reason for its lack of inhibition of OXA enzymes seems to be related to availability of hydrogen bond donors. In class A enzymes, there are hydrogen bond donors that interact with the amide carbonyl, while OXA enzymes lack these donors. Instead, the corresponding region is hydrophobic and quite enclosed in class D (22). For vaborbactam, this results in unfavorable contacts, diminishing activity against OXA enzymes. QPX7728 lacks this amide and apparently avoids this problem with a resulting increase in potency toward OXA enzymes.

**TABLE 1:** IC_50_ values (in nM) of β-lactamase inhibition by QPX7728 and comparator BLIs^*b*^

Enzyme	Class	CARB	IC_50_ (nM) of drug:			
			Vaborbactam	Avibactam	Relebactam	QPX7728
KPC-2	A	Yes	110 ± 30	22 ± 6	82 ± 17	2.9 ± 0.4
CTX-M-14	A	No	110 ± 40	1.4 ± 0.4	34 ± 10	0.94 ± 0.2
CTX-M-15	A	No	92 ± 13	0.56 ± 0.25	ND	1.2 ± 0.1
SHV-12	A	No	56 ± 11	0.61 ± 0.19	330 ± 50	1.9 ± 0.6
TEM-10	A	No	470 ± 150	4.3 ± 1.2	160 ± 20	2.2 ± 0.8
TEM-43	A	No	(2.2 ± 0.4) × 10^3^	ND	ND	3.0 ± 0.1
P99	C	No	88 ± 38	26 ± 7	36 ± 4	22 ± 6
OXA-48	D	Yes	(6.9 ± 2.3) × 10^3^	180 ± 50	(9 ± 0.3) × 10^4^	1.1 ± 0.4
OXA-23	D	Yes	(1.2 ± 0.2) × 10^5^	(3.1 ± 0.6) × 10^3^	ND	1.2 ± 0.4
NDM-1^*a*^	B	Yes	>1.6 × 10^5^	>1.6 × 10^5^	>1.6 × 10^5^	55 ± 25
VIM-1	B	Yes	>1.6 × 10^5^	>1.6 × 10^5^	>1.6 × 10^5^	14 ± 4
IMP-1^*a*^	B	Yes	>1.6 × 10^5^	>1.6 × 10^5^	>1.6 × 10^5^	610 ± 70
IMP-26^*a*^	B	Yes	>1.6 × 10^5^	>1.6 × 10^5^	>1.6 × 10^5^	(4.1 ± 1) × 10^3^

a Imipenem was used as a substrate.

b Abbreviations: CARB, carbapenemase activity; ND, not done.

Notably, QPX7728 inhibited clinically important MBLs with considerable potency. VIM-1 and NDM-1 were inhibited with IC_50_ values ranging from 14 nM to 55 nM. Somewhat lower potency was observed for MBLs from the IMP subgroup, 0.61 μM and 4 μM for IMP-1 and IMP-26, respectively (Table 1). As expected, none of the comparator BLIs had any MBL-inhibitory activity (IC_50_, >160 μM).

MBLs that are inhibited by QPX7728 belong to the B1 subclass of the class B family; this subclass contains the most clinically relevant beta-lactamases, such as NDM and VIM (8). Two other subclasses, B2 and B3, which represent less common enzymes, differ from B1 based on sequence and structure (in particular, B3), amino acid residues involved in Zn coordination, and even the number of Zn atoms involved in catalysis. No QPX7728 inhibition experiments have been performed with purified B2 and B3 MBLs to date. However, microbiological experiments demonstrated the inability of QPX7728 to reduce MICs of meropenem and ceftazidime against the strain producing cloned L1 from Stenotrophomonas maltophilia, the MBL from the B3 subclass (23), suggesting that QPX7728 is a poor inhibitor of L1. This suggests that a different structural scaffold may be required for interaction with this group of MBLs.

### QPX7728 is a covalent slowly reversible inhibitor of serine beta-lactamases.

**FIG 2:**
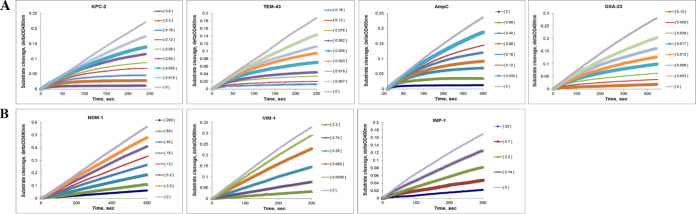
Kinetic profiles of inactivation of serine and metallo-beta-lactamases by QPX7728. (A) Serine beta-lactamases; (B) metallo beta-lactamases. QPX7728 at indicated micromolar concentrations was quickly mixed with each enzyme and 100 μM nitrocefin, and absorbance at 490 nm was recorded immediately every 2 s using a plate reader.

The kinetic mechanism of QPX7728 interaction with serine and metallo-beta-lactamases was studied using nitrocefin, and several purified enzymes (kinetic parameters for all the beta-lactamases used in these studies are presented in Table S1 in the supplemental material). The profiles of inactivation of beta-lactamase activity by QPX7728 for selected enzymes are presented in Fig. 2a. For all the tested serine beta-lactamases, QPX7728 demonstrated biphasic inactivation kinetics with a slow onset of inhibition and nonlinear reaction profiles characteristic of progressive inactivation of activity. Additionally, this kinetic behavior is manifested by the decrease of apparent *K_i_* values (*K_i_*_app_) when enzyme and BLI are preincubated for various times (Fig. S1). This type of kinetics is described by the initial formation of a noncovalent complex EI (characterized by binding constant *K*) that subsequently proceeds to a covalent interaction between the catalytic serine residue of the enzyme and a boron atom of a BLI in the EI* complex (24). This second step is described by the first-order rate constant *k*_2_.

$$\text{E + I}\overset{K}{↔}\text{EI}\overset{k_{2}}{\underset{k_{-2}}{↔}}{\text{EI}}^{*}$$

Enzyme inactivation efficiency is defined by the second-order constant *k*_2_/*K* which was determined for several class A, class C, and class D beta-lactamases (Table 2). QPX7728 efficiently inactivated all tested beta-lactamases: the lowest *k*_2_/*K* was for the class C P99 enzyme and was still of an appreciable value of ∼6 × 10^4^ M^−1^ s^−1^. For class A beta-lactamases, the inactivation constant *k*_2_/*K* of QPX7728 varied over a 10-fold range with the lowest and the highest inactivation efficiency of 1.0 × 10^5^ M^−1^ s^−1^ and 1.8 × 10^6^ M^−1^ s^−1^ for SHV-12 and BKC-1, respectively. *k*_2_/*K* for KPC-2 and KPC-3 were 3 × 10^5^ to 4 × 10^5^ M^−1^ s^−1^, which is almost 100-fold higher than that for vaborbactam. QPX7728 inhibited all tested class D carbapenemases with a high efficiency: *k*_2_/*K* for OXA-48 from *Enterobacteriaceae* was ∼3 × 10^6^ M^−1^ s^−1^, and *k*_2_/*K* for OXA carbapenemases from A. baumannii (OXA-23, OXA-24, and OXA-58) was ∼1 × 10^6^ M^−1^ s^−1^.

**TABLE 2:** Kinetic parameters of beta-lactamase inactivation by QPX7728

Enzyme	*k*_2_/*K* (M^−1^ s^−1^)	*k*_off_, s^−1^	Residence time, min	*K_d_*, nM^*a*^	Stoichiometry
KPC-2	(3.6 ± 0.1) × 10^5^	(9.0 ± 1.4) × 10^−5^	189 ± 31	0.25 ± 0.03	1
KPC-3	(4.1 ± 1.0) × 10^5^	(1.26 ± 0.07) × 10^−4^	133 ± 8	0.31 ± 0.06	1
BKC-1	(1.82 ± 0.04) × 10^6^	(1.1 ± 0.1) × 10^−4^	154 ± 14	0.060 ± 0.005	1
FRI-1	(1.15 ± 0.03) × 10^6^	(1.2 ± 0.2) × 10^−4^	138 ± 24	0.11 ± 0.01	1
SME-2	(1.2 ± 0.1) × 10^6^	(1.8 ± 0.2) × 10^−4^	94 ± 11	0.15 ± 0.02	1
CTX-M-15	(6.9 ± 0.6) × 10^5^	(8.0 ± 1.0) × 10^−5^	220 ± 33	0.11 ± 0.02	1
SHV-12	(1.1 ± 0.2) × 10^5^	(3.0 ± 0.2) × 10^−3^	5.5 ± 0.3	28 ± 4	1
TEM-43	(1.9 ± 0.3) × 10^6^	(3.2 ± 0.2) × 10^−4^	53 ± 3	0.17 ± 0.02	1
P99	(6.3 ± 0.7) × 10^4^	(3.3 ± 0.3) × 10^−5^	506 ± 51	0.53 ± 0.06	1
OXA-48	(2.75 ± 0.09) × 10^6^	(3.6 ± 0.2) × 10^−4^	47 ± 3	0.13 ± 0.01	1
OXA-23	(9.9 ± 0.6) × 10^5^	(1.6 ± 0.2) × 10^−3^	11 ± 2	1.6 ± 0.2	2
OXA-24	(1.5 ± 0.2) × 10^6^	(9.0 ± 1.0) × 10^−4^	20 ± 3	0.58 ± 0.10	1
OXA-58	(1.07 ± 0.08) × 10^6^	(3.5 ± 0.3) × 10^−4^	4.8 ± 0.5	3.2 ± 0.3	1
NDM-1	ND^*b*^	ND	ND	32 ± 14	ND
VIM-1	ND	ND	ND	7.5 ± 2.1	ND
IMP-1	ND	ND	ND	240 ± 30	ND

a *K_d_* values for serine beta-lactamases were derived from the ratio of *k*_off_ and *k*_2_/*K. K_d_* values for MBLs were based on *K_i_*s.

b ND, not done.

The crystal structures of several serine beta-lactamases, CTX-M-14, KPC-2, and OXA-48, bound to QPX7728 were recently determined (20). In these enzymes, the catalytic serine residue is covalently bound to the boron atom of the inhibitor, validating the applicability of the kinetic model that was used to determine the inactivation efficiency of QPX7728.

**FIG 3:**
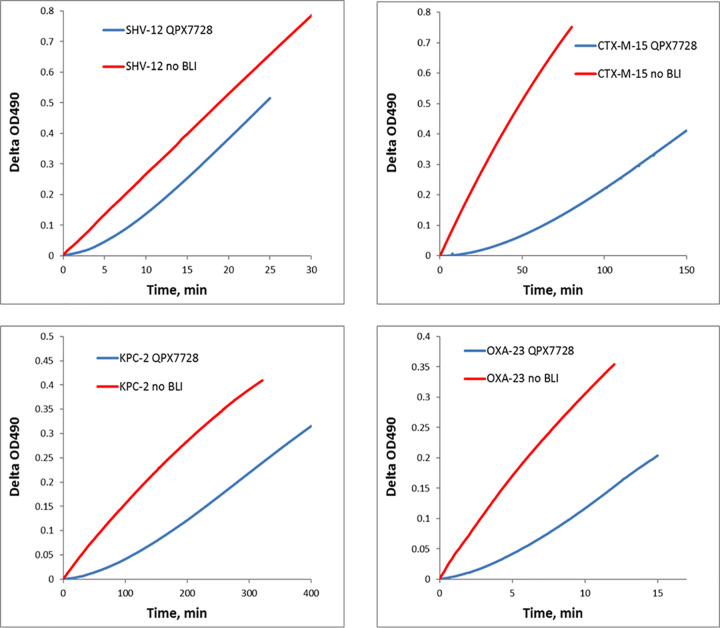
Kinetic profiles of activity recovery of various beta-lactamases after inhibition by QPX7728 using the jump dilution method. Enzymes at a 1 μM concentration were mixed with QPX7728 at an 8 μM concentration and incubated for 30 min. After appropriate dilution, 200 μM nitrocefin was added to the reaction mixture and absorbance at 490 nm was recorded every 10 s using a plate reader. A reaction without addition of BLI was also recorded and used to calculate uninhibited enzyme velocity *V_s_*.

The reversibility of inhibition of serine beta-lactamases by QPX7728 was evaluated by the “jump dilution” method as previously described for avibactam (15) and vaborbactam (17). QPX7728 produced typical enzyme recovery profiles characteristic of reversible inhibition (Fig. 3 shows some of the recovery curves). *k*_off_ values for different beta-lactamases varied significantly (Table 2). For class A beta-lactamases, they ranged from 0.003 s^−1^ for SHV-12 to 0.00008 s^−1^ for CTX-M-15. That translates to QPX7728 residence time values of 5 min and 220 min, respectively. The residence time for KPC-2/KPC-3 was similar to that of CTX-M-15, 130 to 190 min. A low *k*_off_ value of 0.000033 s^−1^ with a residence time of ∼500 min was determined for P99 inhibition by QPX7728. Finally, among class D carbapenemases the lowest *k*_off_ values of ∼0.0004 s^−1^ and the longest residence time of 47 min were observed for OXA-48. *k*_off_ and residence time values for OXA carbapenemases from A. baumannii were in the 0.001 to 0.004 s^−1^ range and 5- to 20-min range, respectively.

QPX7728 had 3-fold- to 150-fold-lower *k*_off_ values and longer residence time with most enzymes than vaborbactam. The only exceptions were KPC-2 and KPC-3 (17), for which vaborbactam demonstrated more stable complex formation than QPX7728 with 2- to 3-fold-lower *k*_off_ values. The detailed analysis of the recently solved structures of vaborbactam and QPX7728 bound to KPC is in progress and might provide the structural basis for the observed differences.

Nevertheless, given ca. 100-fold-higher inactivation efficiency of KPC by QPX7728 than by vaborbactam, QPX7728 *K_d_* (dissociation constant) values for KPC-2 and KPC-3 were in a subnanomolar range that was 10- to 50-fold lower than that of vaborbactam (Table 2) (17).

Overall, QPX7728 *K_d_* values for various beta-lactamases ranged from 0.06 nM to 28 nM and from 0.13 nM to 3.2 nM for class A and class D enzymes, respectively. *K_d_* for P99 was 0.59 nM (Table 2). QPX7728 inhibited all tested serine enzymes at a 1:1 molar ratio (Table 2).

### QPX7728 is a “fast-on–fast-off” inhibitor of MBLs.

A very different kinetic inhibition profile by QPX7728 was observed for the metallo-beta-lactamases NDM-1, VIM-1, and IMP-1 (Fig. 2B): QPX7728 produced linear inactivation profiles, with no sign of progressive inactivation indicative of the quickly established equilibrium between the enzyme and an inhibitor which is typical for “fast-on–fast-off” reversible inhibitors. Also, no differences in the QPX7728 *K_i_*_app_ values were observed with variation in the preincubation times of QPX7728 with these MBLs (Fig. S1), in contrast to KPC-2 enzyme, supporting their reversible inhibition by QPX7728. Fast and reversible inhibition is consistent with a noncovalent complex that QPX7728 forms with NDM-1 and VIM-1 based on available structural information (20).

**FIG 4:**
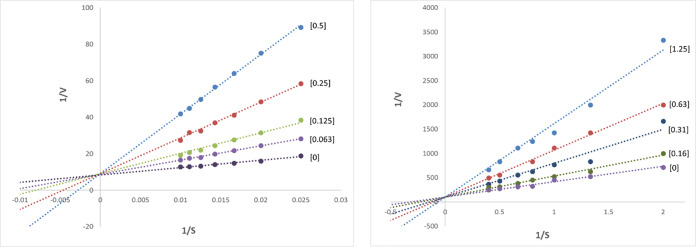
Lineweaver-Burk plots of VIM-1 (left) and NDM-1 (right) inhibition by QPX7728. Enzymes were mixed with various concentrations of nitrocefin substrate (*x* axis) and indicated micromolar amounts of QPX7728 (in brackets), and reaction profiles were recorded for 10 min at 490 nm. Initial rates of reaction were calculated, and corresponding reciprocal values were plotted against 1/*S*.

Further evaluation revealed that QPX7728 behaved as a competitive inhibitor of MBLs (Fig. 4) with *K_i_*s of 7.5 nM, 32 nM, and 240 nM, for VIM-1, NDM-1, and IMP-1, respectively. Given the “fast-on–fast-off” reversible inhibition mechanism, these *K_i_*s can be considered an equivalent of *K_d_* values of QPX7728 for MBLs (Table 2).

QPX7728 has the broadest spectrum of beta-lactamase inhibition compared to the marketed BLIs avibactam, relebactam, and vaborbactam. These marketed agents have activity against class A beta-lactamases including carbapenemases such as KPC, class C beta-lactamases, and some class D enzymes (in the case of avibactam). QPX7728 has two main improvements in spectrum: it is an efficient inhibitor of class D carbapenemases from A. baumannii, such as OXA-23, OXA-24/40, and OXA-58, and it inhibits various class B metallo-beta-lactamases from the B1 subclass such as NDM, VIM, IMP, and others (but not the B3 subclass, such as L1 from S. maltophilia).

Two other investigational BLIs, the avibactam derivative DBO durlobactam (ETX2514) and the bicyclic boronate taniborbactam (VNRX-5133) are in clinical trials (11). Durlobactam is a potent inhibitor of class A, class C, and class D beta-lactamases including those from A. baumannii (25, 26). Based on comparison with published data (16, 25, 26), QPX7728 and durlobactam have similar *K_d_*s for OXA carbapenemases from A. baumannii which are in the low-nanomolar range. But unlike QPX7728, durlobactam does not inhibit metallo-beta-lactamases.

Taniborbactam inhibits both serine and metallo-beta-lactamases. Based on published data, QPX7728 and taniborbactam appear to have similar potencies of inhibition of serine beta-lactamases and MBLs NDM and VIM. In contrast, QPX7728 is a more potent IMP inhibitor than taniborbactam. Unlike QPX7728, taniborbactam does not inhibit class D carbapenemases from A. baumannii (27–29).

**FIG 5:**
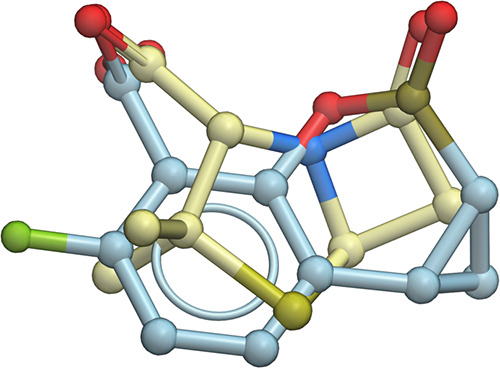
Superposition of QPX7728 and the typical core ring system of beta-lactam antibiotics. QPX7728 in light blue carbon atoms and the typical core ring system of beta-lactam in crème carbon atoms. Close correspondence of molecular volumes and other features between the inhibitor and the substrate core can be observed. Molecular alignment and visualization performed in ICM-Pro (Molsoft, San Diego, CA).

We speculate that the exceptional breadth of the inhibition spectrum of QPX7728 is likely, at least in part, due to its very compact structure—the inhibitor is barely larger than the 5/4 core ring system of the beta-lactam substrates (Fig. 5) and fits entirely within the immediate vicinity of the active site. Lack of any side chains extending into more distal regions allows the inhibitor to avoid potentially unfavorable interactions that could arise due to sequence/structure variations. Notably, the expansion of spectrum achieved in QPX7728 did not come at the expense of reduced potency against the class A and class C beta-lactamases; for example, QPX7728 has ca. 8- to 30-fold-higher potency against KPC-2 than do avibactam, relebactam, and vaborbactam and appears to have similar potency against class C P99.

We speculate that one significant factor enhancing the potency of QPX7728 compared to vaborbactam is its near-total rigidity. Thus, conformational entropy loss upon binding is negligible for QPX7728. In contrast, vaborbactam has 5 rotatable bonds and, as evidenced by X-ray structures (12), a ring with two alternative low-energy conformations, a total of 6 degrees of freedom. The entropic penalty upon binding for each lost degree of conformational freedom might result in a significantly increased *K_d_*_._: a ballpark estimate of 0.6 kcal/mol per degree of freedom would give a 3.6-kcal/mol free energy penalty, equivalent to a several-hundredfold difference in *K_d_*.

QPX7728 demonstrates progressive inactivation of all serine beta-lactamases, and its inactivation efficiency is generally very high. For example, QPX7728 inactivates KPC-2 (*k*_2_/*K* of 3.6 × 10^5^ M^−1^ s^−1^) ca. 10-fold more efficiently than avibactam (*k*_2_/*K* of 1.3 × 10^4^ (14) and relebactam (*k*_2_/*K* of 2.5 × 10^4^ [30]) and almost 100-fold more efficiently than vaborbactam (*k*_2_/*K* of 5.5 × 10^3^ [17]). In addition to the high inactivation efficiency, QPX7728 forms a very tight complex with some serine beta-lactamases with calculated residence times reaching several hours. In the case of KPC-2, the residence time of QPX7728 is 3 h. This is comparable to ∼2 h for avibactam (14), 1.5 h for relebactam (30), and ∼6 h for vaborbactam (17). Thus, QPX7728 is a covalent low-off-rate inhibitor of serine enzymes. In contrast, inhibition of metallo-beta-lactamases by QPX7728 (similar to taniborbactam [27]) proceeds through a simple one-step complex formation typical for fast-on–fast-off inhibitors.

### Conclusions.

QPX7728 has an ultrabroad spectrum of beta-lactamase inhibition. It has two major improvements in spectrum over the currently marketed agents (avibactam, vaborbactam, and relebactam) and clinical-stage investigational agents (durlobactam and taniborbactam): it is an efficient inhibitor of class D carbapenemases from A. baumannii, such as OXA-23, OXA-24/40, and OXA-58, and it inhibits various class B metallo-beta-lactamases from the B1 subclass, such as NDM, VIM, and IMP.

## MATERIALS AND METHODS

### Beta-lactamase enzyme preparations.

All purified beta-lactamase enzymes used in the study were either expressed and purified internally (17) or obtained from Emerald Biostructures (Bainbridge Island, WA).

### Determination of IC_50_ and *K_i_* values of beta-lactamase inhibition by BLI with nitrocefin or imipenem as a substrate.

Enzymes were mixed with BLIs at concentrations varying from 160 to 0.0027 μM in 50 mM sodium phosphate (pH 7.0)-0.1 mg/ml bovine serum albumin (BSA) (buffer A; 20 μM ZnCl_2_ was also added for all metallo enzymes) and incubated for 10 min at 37°C. A 50 μM concentration of nitrocefin (10 μM for SHV-12) or 100 μM imipenem (prewarmed at 37°C for 10 min) was added, and substrate cleavage profiles were recorded at 37°C at 490 nm every 10 s for 10 min or at 294 nm every 30 s for 1 h for nitrocefin and imipenem, respectively. Initial rates of reaction were calculated and exported to Prism software to calculate IC_50_ values using the “dose-response—inhibition, variable slope (four parameters)” equation. *K_i_* values were calculated by the method of Waley (31). This method was previously used to calculate *K_i_* values for boronic fast-on–fast-off BLIs.

### Determination of *k*_2_/*K* inactivation constant for various enzymes.

Inactivation kinetic parameters were determined by the reporter substrate method (32) for the slow-tight binding inhibitor kinetic scheme.

$$\text{E + I}\overset{K}{↔}\text{EI}\overset{k_{2}}{\underset{k_{-2}}{↔}}{\text{EI}}^{*}$$

Enzyme was quickly mixed with 100 μM nitrocefin (NCF) and various concentrations of inhibitors in reaction buffer, and absorbance at 490 nm was measured immediately every 2 s for 600 s on a SpectraMax plate reader (Molecular Devices) at 490 nm. Resulting progression curves of optical density at 490 nm (OD_490_) versus time at various BLI concentrations were imported into Prism software (GraphPad), and pseudo-first-order rate constants *k*_obs_ were calculated using the following equation:

$$P=V_{s}\times \left(\text{1}-e^{-k_{\text{obs}}}{}^{\times t}\right)/k_{\text{obs}}$$

where *V_s_* is uninhibited enzyme rate. *k*_obs_ values calculated at various BLI concentrations were fitted in the following equation: *k*_obs_ = *k*_−2_ + *k*_2_/*K* × [*I*]/(1 + [NCF]/*K_m_*, NCF), where *k*_2_/*K* is inactivation constant, [*I*] is inhibitor concentration, [NCF] is nitrocefin concentration, and *K_m_*, NCF is Michaelis constant of NCF for a given enzyme.

### Stoichiometry of beta-lactamase inhibition by BLIs.

Purified enzyme at 1 μM concentration in buffer A was mixed with BLI at a BLI/enzyme ratio varying from 128 to 0.0625. After 30 min of incubation at room temperature, the reaction mixture was diluted 100-fold, and enzyme activity was measured with nitrocefin as described above. Stoichiometry of inhibition was determined as a minimal BLI/enzyme ratio reducing enzyme activity by at least 90%.

### Determination of *k*_off_ rates of enzyme activity recovery after inhibition by BLIs.

Enzyme at a 1 μM concentration in buffer A was mixed with BLI at an 8-fold-higher concentration than its stoichiometry ratio (determined in preliminary stoichiometry experiments). After 30 min of incubation at 37°C, the reaction mixture was diluted from 1,000-fold to 10,000-fold (depending on enzyme) in buffer A, and 100 μl of diluted enzyme was mixed with 100 μl of 400 μM nitrocefin in reaction buffer. Absorbance at 490 nm was recorded for 4 h each minute at 37°C. Resulting reaction profiles were fitted into the following equation using Prism software (GraphPad) to obtain *k*_off_ values: *P* = *V_s_* × *t* + (*V_o_* − *V_s_*) × (1 − *e*(−*k*_off_ × *t*))/*k*_off_, where *V_s_* is uninhibited enzyme velocity, measured in the reaction with enzyme and no inhibitor, and *V_o_* is completely inhibited enzyme velocity, measured in the reaction with no enzyme and nitrocefin only.

### Statistical analysis.

All kinetic results are presented as average ± standard deviation for a minimum of three replicates.

## Supplementary Material

Supplemental file 1
