# Supplementary material for: Biochemical Characterization of QPX7728, a New Ultrabroad-Spectrum Beta-Lactamase Inhibitor of Serine and Metallo-Beta-Lactamases
Source: Antimicrob Agents Chemother. 2020 May 21;64(6):e00130-20. doi: 10.1128/AAC.00130-20 (PMC7269513; doi:10.1128/AAC.00130-20)
Supplement: Supplemental file 1 [file AAC.00130-20-s0001.pdf]

Supplementary Table S1

Table S1. K_m and k_{cat} values of nitrocefin or imipenem hydrolysis for all enzymes used in the study

Enzyme	Substrate	K_m, µM	k_{cat}, sec^{-1}	k_{cat}/K_m, $sec^{-1}*\mu M^{-1}$
CTX-M-14	Nitrocefin	22	334	15
CTX-M-15	Nitrocefin	24	341	15
SHV-12	Nitrocefin	5.5	73	13
TEM-10	Nitrocefin	16	18	1.1
TEM-43	Nitrocefin	44	175	4.0
P99 (AmpC)	Nitrocefin	100	479	4.8
KPC-2	Nitrocefin	49	206	4.3
KPC-2	Imipenem	70	39	0.6
KPC-3	Nitrocefin	49	182	3.7
OXA-48	Nitrocefin	29	406	14
OXA-48	Imipenem	1.5	1.7	1.1
BKC-1	Nitrocefin	9.5	721	78
FRI-1	Nitrocefin	75	176	2.4
SME-2	Nitrocefin	59	352	6
OXA-23	Nitrocefin	62	357	5.8
OXA-23	Imipenem	6.6	0.7	0.11
OXA-24	Nitrocefin	28	208	7.6
OXA-24	Imipenem	1.2	1.9	1.7
OXA-58	Nitrocefin	34	518	15
OXA-58	Imipenem	1.3	0.66	0.51
VIM-1	Nitrocefin	55	133	2.4
VIM-1	Imipenem	13	15	1.1
NDM-1	Nitrocefin	1.6	27	17
NDM-1	Imipenem	206	96	0.46
IMP-1	Nitrocefin	26	229	8.9
IMP-1	Imipenem	66	35	0.52
IMP-26	Nitrocefin	81	325	4.0
IMP-26	Imipenem	64	35	0.55

Supplementary Figure S1. $K_{i\ app}$ (or K_i) values of KPC-2 and NDM-1 inhibition by QPX7728 after various preincubation times.

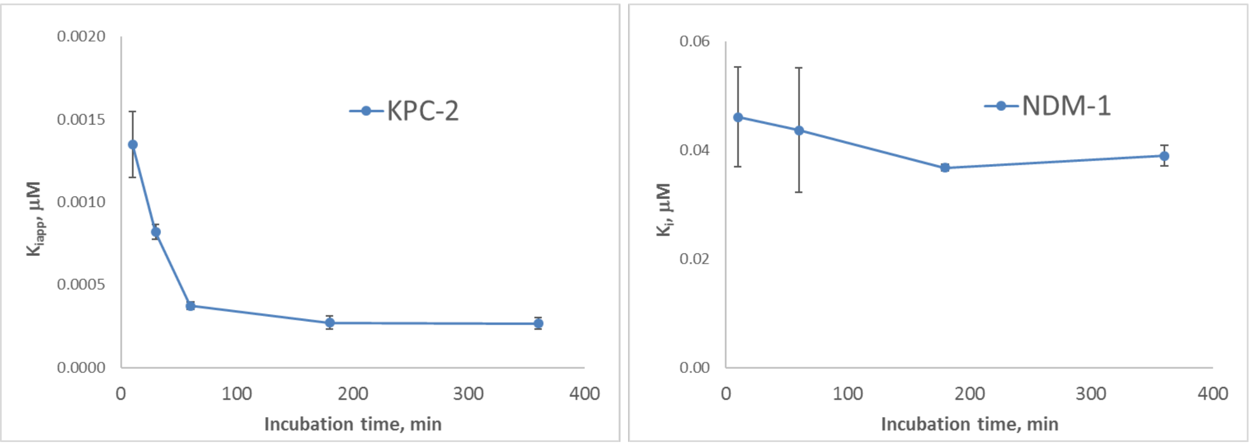

KPC-2 and NDM-1 enzymes were mixed with wide range of concentrations of QPX7728 and incubated at various times indicated on X axis. Next, 50 µM nitrocefin (KPC-2) or 100 µM imipenem (NDM-1) was added and reaction profiles were recorded for 10 minutes at 490 and 294 nm, respectively. $K_{i\ app}$ and K_i values were calculated as described in (1).

References

1. Waley SG. 1982. A quick method for the determination of inhibition constants. Biochem J 205:631-3.
